# Imaging Flow Cytometry for High-Throughput Phenotyping of Synthetic Cells

**DOI:** 10.1021/acssynbio.3c00074

**Published:** 2023-05-08

**Authors:** Elisa Godino, Ana Maria Restrepo Sierra, Christophe Danelon

**Affiliations:** † Department of Bionanoscience, Kavli Institute of Nanoscience, Delft University of Technology, 2629HZ Delft, The Netherlands; ‡ Toulouse Biotechnology Institute (TBI), Université de Toulouse, CNRS, INRAE, INSA, 31077 Toulouse, France

**Keywords:** synthetic cell, minimal cell, cell-free expression, liposome, in vitro transcription−translation, directed evolution

## Abstract

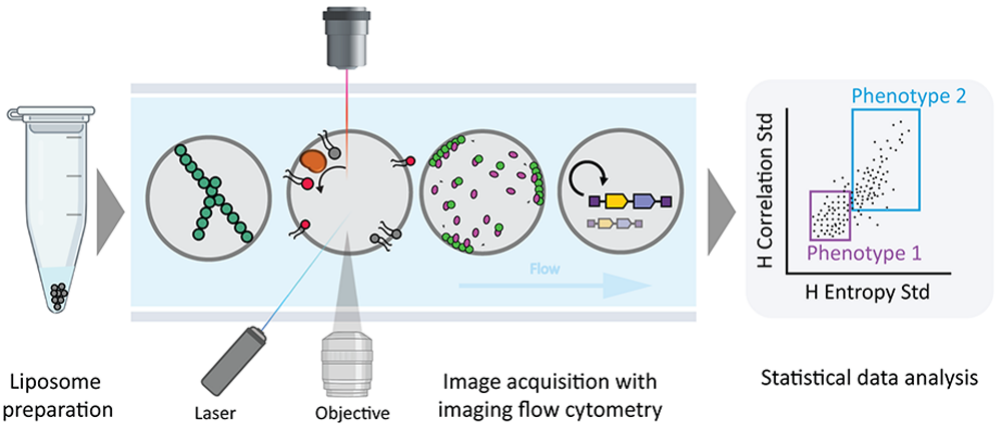

The reconstitution of basic cellular functions in micrometer-sized liposomes has led to a surge of interest in the construction of synthetic cells. Microscopy and flow cytometry are powerful tools for characterizing biological processes in liposomes with fluorescence readouts. However, applying each method separately leads to a compromise between information-rich imaging by microscopy and statistical population analysis by flow cytometry. To address this shortcoming, we here introduce imaging flow cytometry (IFC) for high-throughput, microscopy-based screening of gene-expressing liposomes in laminar flow. We developed a comprehensive pipeline and analysis toolset based on a commercial IFC instrument and software. About 60 thousands of liposome events were collected per run starting from one microliter of the stock liposome solution. Robust population statistics from individual liposome images was performed based on fluorescence and morphological parameters. This allowed us to quantify complex phenotypes covering a wide range of liposomal states that are relevant for building a synthetic cell. The general applicability, current workflow limitations, and future prospects of IFC in synthetic cell research are finally discussed.

## Introduction

Synthetic lipid vesicles, called liposomes, are widely used as biological membrane models for basic and applied research.^1,2^ By virtue of their biocompatibility, nonimmunogenicity, and easy manufacturing, liposomes are successfully employed as pharmaceutical (nano)carriers.^3^ Moreover, they are routinely utilized as bioreactors, diagnostic and biosensing tools, and as a proxy of cellular membranes to study a variety of biochemical and biophysical mechanisms.^2^ In particular, giant vesicles with a diameter typically >1 μm provide a versatile cell-like platform for the reconstitution of various biological processes, such as DNA replication,^4^ cytokinesis using prokaryotic or eukaryotic protein systems,^5−9^ dynamic self-organization at the membrane,^7,10^ light-driven ATP production,^11^ and cell–cell communication mimicry.^12^ The extended repertoire of reconstituted biological functions has now prompted synthetic biologists to envision the construction of an entire synthetic cell starting from liposomes as the chassis.

A major challenge to engineering artificial cells with advanced functionalities lies in the ability to detect complex phenotypes (hereafter referring to any measurable properties, e.g., protein concentration, localization, and liposome shape) in large populations of liposomes. This is critical to identify vesicles exhibiting desired features from heterogeneous pools, as well as to optimize and compare protocols for the production of liposomes on the basis of quantifiable parameters (yield, size homogeneity, lamellarity, activity of internalized components). Appropriate analytical methods therefore need to generate information-rich data from individual liposomes for accurate phenotype identification, combined with high-speed screening for massive data collection from large populations of vesicles.

Owing to their large size, giant vesicles are regularly imaged at high spatial and temporal resolution by fluorescence microscopy techniques. Dynamical behaviors, such as biochemical pattern formation, membrane fluctuations, and morphological changes can be visualized in real time at the single vesicle level. However, fluorescence imaging generally suffers from a low screening capability, and it remains challenging to convert single-liposome properties into a large data set that enables extraction of rare phenotypes in a quantitative and statistically relevant manner.^13^ On the other hand, flow cytometry is a powerful technology for high-throughput screening of giant vesicles.^13,14^ Individual liposomes (or aggregates) diluted in suspension are sequentially analyzed based on scattered light and fluorescence intensity signals. One drawback of flow cytometry is that data rely on a one-dimensional fluorescence signal, which severely limits the spectrum of features that can be investigated.

To alleviate the limitations inherent to conventional fluorescence microscopy and flow cytometry for high-throughput interrogation of complex liposome phenotypes, we propose here to combine their strengths by using imaging flow cytometry (IFC). By enabling the rapid acquisition of multispectral images of single cells in flow, IFC has gained popularity in a variety of cell biology-related disciplines, where the identification and quantification of rare cellular phenotypes within heterogeneous populations are important.^15^ For example, IFC has been instrumental to study apoptosis in relation to alterations of nuclear morphology and structure,^16^ cell cycle progression based on chromatin condensation,^17^ protein and molecule translocation and/or colocalization in different cellular compartments,^18,19^ and cytoskeleton structures.^20^ To our knowledge, IFC has been applied to giant vesicle suspensions in only one study that focused on the optimization protocol of liposome production with water-in-oil emulsion transfer methods.^21^

**Figure 1:**
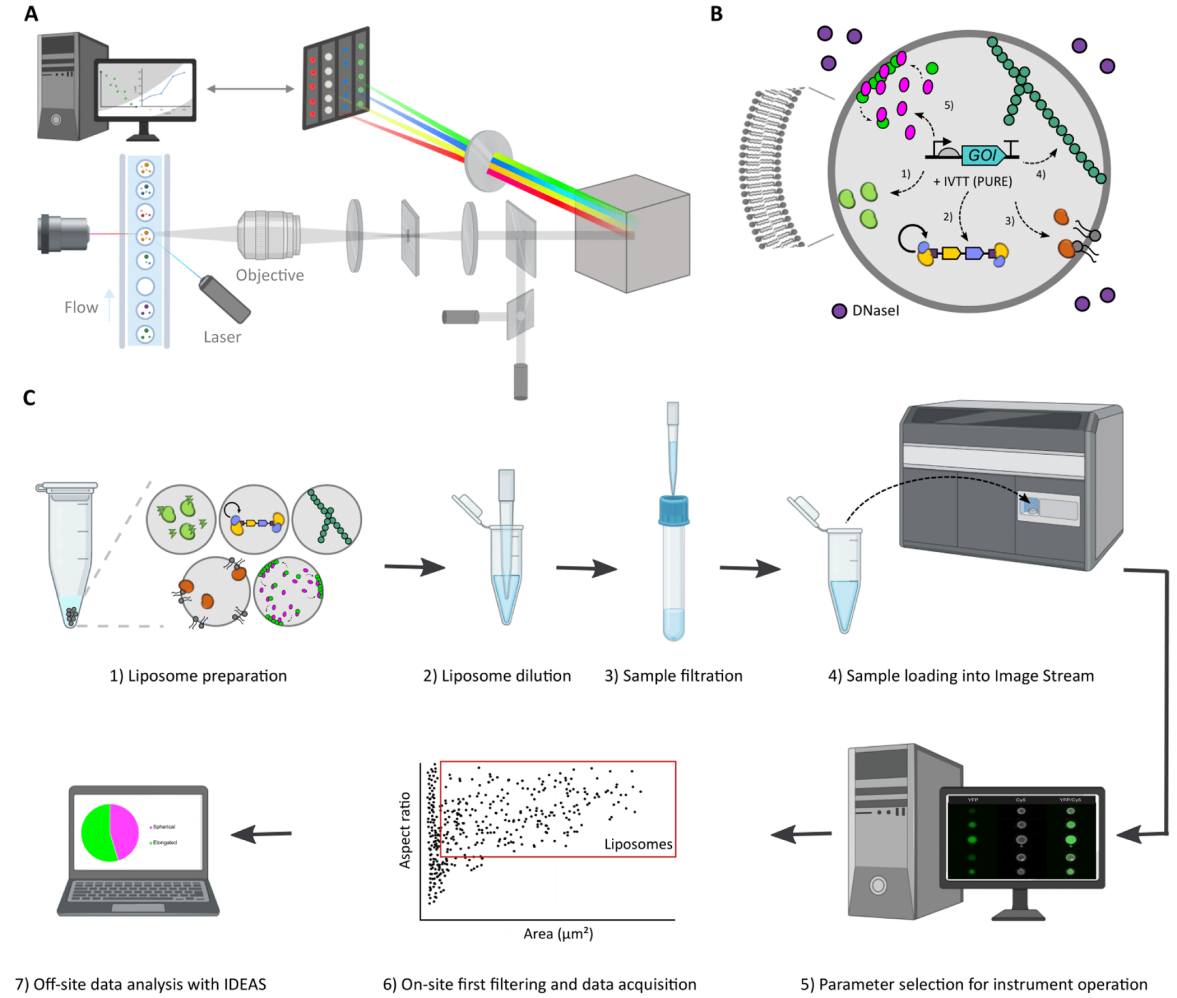
Overview of the IFC workflow to characterize gene expressing liposomes. (A) Schematic illustration of the primary components of ImageStream. Giant vesicles are hydrodynamically focused into a core stream and orthogonally illuminated. The emitted or scattered light is captured by the imaging objective, separated into multispectral bands via optical decomposition components, and projected on a charge-coupled detector. While recording, digital pictures are displayed on the computer and stored for analysis. Image adapted with permission from ref (16). Copyright (2004) John Wiley and Sons. (B) Illustration of a liposome-based synthetic cell containing relevant biological processes that have separately been expressed from genes with PURE system: transcription–translation (1), DNA replication (2), phospholipid biosynthesis (3), and the formation of cytoskeletal structures (4). (C) Overview of the workflow for sample treatment, data acquisition, and image processing.

In this work, we leverage the commercial IFC instrument ImageStream (Luminex Corporation) to characterize synthetic cell modules from liposome populations (Figure 1A,B). ImageStream is a benchtop imaging flow cytometer that enables multispectral acquisition of individual cells or objects in flow. We develop a comprehensive pipeline for liposome identification, selection of unilamellar vesicles, and multimodal analysis of image-based phenotypes using the built-in image processing software IDEAS. We finally discuss the current limitations and opportunities of IFC as an enabling technology to accelerate synthetic cell research.

## Results

### Liposome Detection Pipeline

Samples consisted of phospholipid vesicles containing PURE system, a reconstituted transcription–translation machinery,^22,23^ to support gene expression in synthetic cells (Figure 1B). In most experiments, a small fraction of fluorescently labeled phospholipids was included in the membrane composition for imaging. The glass bead-assisted lipid film swelling method was employed for liposome production (see Methods section) as it proved successful for expression of various cellular modules with PURE system. We collected for each sample around a million events, which were analyzed in a sequential batch mode by opening 100,000 recorded items at a time in IDEAS (Figure 1C). With “events” we here mean any objects whose fluorescence signal in the membrane dye channel crosses the detection threshold. Sample dilution (1 μL liposome stock solution was diluted 100 times) was set to record 800 to 1,000 events per second. With the instrument stabilization and calibration steps, measurement time for one sample was about 2 h.

**Figure 2:**
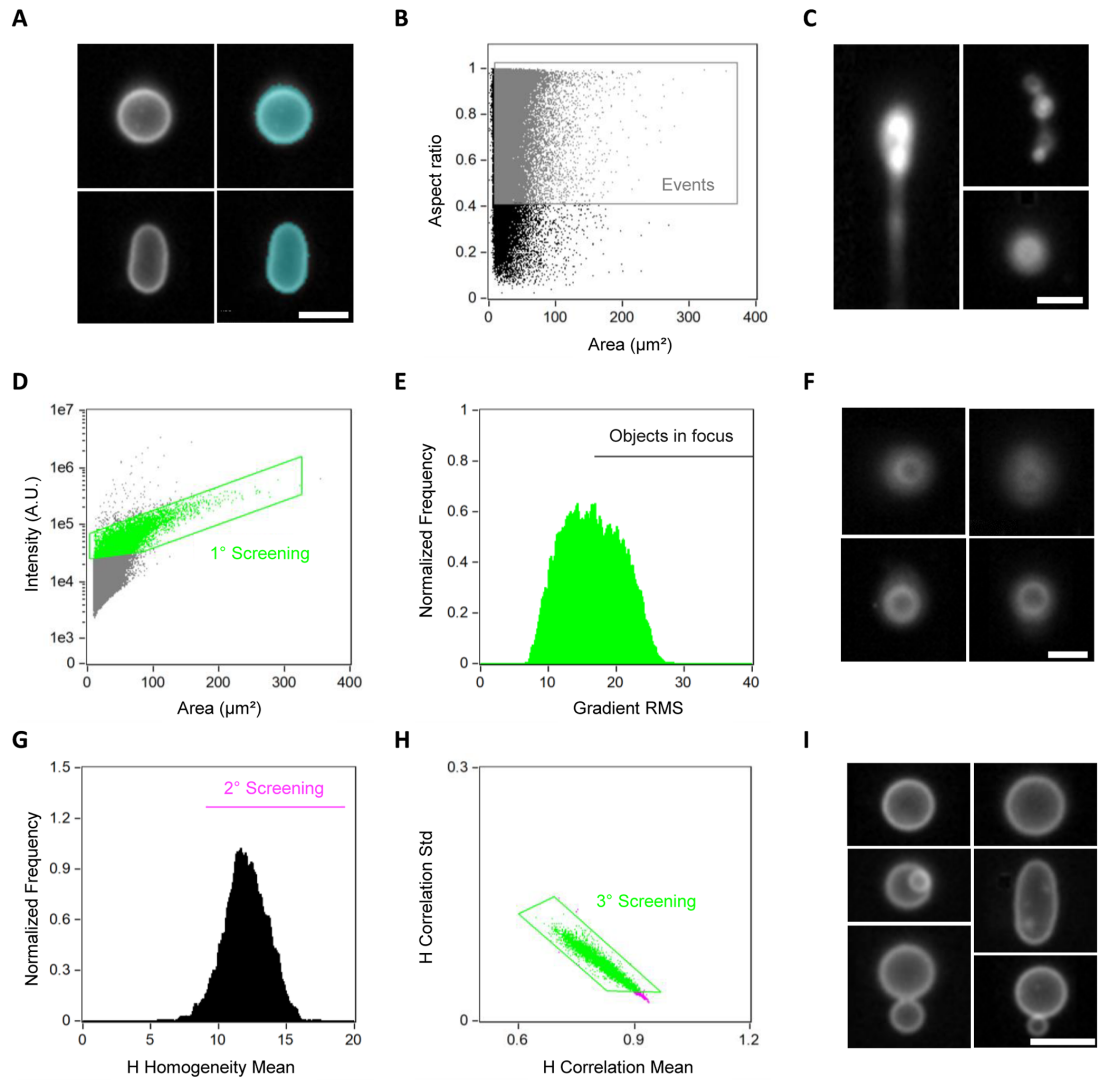
Sequential gating pipeline for identification of liposomes. (A) Images of two liposomes (left) acquired with ImageStream and their respective mask (right). The mask specifies the area to be used during the analysis and it was customized to select for the whole liposome. (B) Data plot showing the *area* and *aspect ratio* features of collected events. Objects having a surface area higher than 8 μm^2^ and an aspect ratio higher than 0.4 were gated as relevant events (in gray). (C) Images of some aggregates with different sizes and shapes that were filtered out by the gating step in (B). (D) Comparison of the *intensity* of the membrane signal vs the *area* of the mask. Selected data points are gated in green. (E) Out-of-focus events were discarded based on the *gradient RMS feature*. Events with a *gradient RMS* higher than 18 were selected for further analysis. (F) Examples of images showing out-of-focus liposomes that were discarded from the analysis. (G) Second gating step aiming at selecting objects with high *H-homogeneity values* (in magenta). (H) Final selection criterion based on the analysis of the *H-correlation mean* and *standard deviation* functions. The selected objects (gated in green) correspond to the final library of liposomes, which is clean from undesired objects. (I) Small gallery of liposome images that passed the gating pipeline. For all the images, the white color represents the membrane dye signal. Scale bars are 7 μm.

We encountered that the raw file contained not only well-defined and isolated liposomes, but also aggregates of all sizes and shapes, lipid debris or small (<300 nm) vesicles, and left-over speed beads (employed to monitor sample flow, and ensure focus and core tracking). Therefore, the first step in the overall analysis pipeline consisted in setting up a gating strategy for selecting solely giant liposomes and excluding any other objects. At every step of the analysis, visual inspection of a subset of the images was carried out to assess the performance of different combinations of features (physical quantities, written in italic) in a particular gating task. The IDEAS software offers a suite of integrated tools for high-content image analysis and data visualization. We started by defining an appropriate mask, i.e., the area of an image that will be used for further processing. Although the IDEAS software includes preloaded masks, it is possible to create customized ones by adjusting the channel and scalar values to better determine the section of the image that will be used for each feature computation. We decided to readjust the default mask to better encompass both the lumen and membrane of individual liposomes (Figure 2A). Then, we performed a comparative analysis over the *area* and *aspect ratio* features (Figure 2B). As the pixels are rendered into square micrometers (μm^2^), the area is given as the microns squared within the utilized mask. We determined that any object having a surface area lower than 8 μm^2^ was not detected with sufficient resolution. The aspect ratio corresponds to the ratio between the minor axis and the major axis of each object and specifies how round an item is. The aspect ratio of circular objects equals one, while oblong structures have significantly lower values. Thus, all objects with an aspect ratio of less than 0.4 were excluded (Figure 2B,C). To further improve the identification of liposomes, we devised an additional three-step screening method for the selection of in-focus events. We first compared the *intensity* of the membrane signal vs the *area* of the mask (Figure 2D). Events with a small area and low membrane intensity were identified as debris or out-of-focus objects and were discarded from the analysis. Objects with a small area and high membrane intensity were discarded as dense lipid aggregates. Then, we used the *gradient RMS* feature to only select in-focus liposomes (Figure 2E). This feature assesses the sharpness of an image by identifying large variations in pixel intensity values. We found that all events with a gradient RMS lower than 18 corresponded to out-of-focus liposomes (Figure 2E,F). Next, we selected objects with high *H-homogeneity* values (Figure 2G), followed by a final step based on the *H-correlation mean* and its *standard deviation* (Figure 2H). These H features establish a set of textures that describe the spatial relationships between the pixel values within the mask. We identified that low H-homogeneity with an elevated H-correlation mean and a low standard deviation relate to aggregates. We confirmed the accuracy of the liposome selection pipeline by visually inspecting a large number of images from the final collection (Figure 2I). As staining liposomes with a membrane dye is not always possible or desired, we showed that a similar sequential gating approach as described above was also applicable to the brightfield images (Figure S1). Overall, from the one million events detected per sample, around 6% were classified as “good” liposomes and were subjected to more advanced image processing to measure morphological features and fluorescence localization.

### Morphometric Analysis of Liposomes

**Figure 3:**
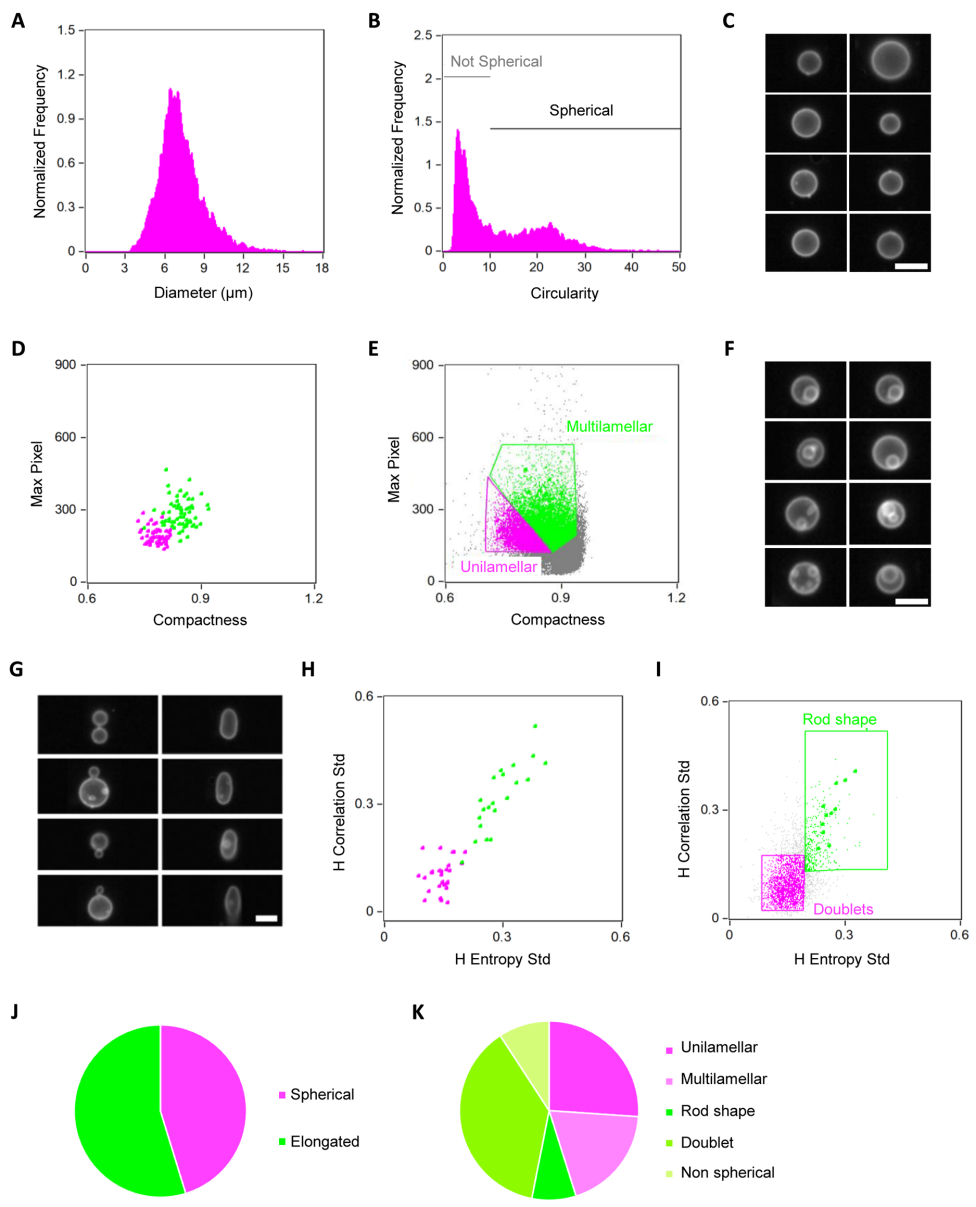
Characterization of liposome morphology. (A) Distribution of liposome diameter. (B) Shape analysis using the *circularity* feature. Objects with a high circularity value were gated as spherical liposomes. (C) Image gallery of some liposomes classified as spherical. (D) Two-dimensional plot of the *compactness* and *max pixel* features as output from the *feature finder wizard* tool performed to distinguish liposomes having intravesicular membrane structures. (E) Population analysis (*compactness* vs *max pixel*) applied to the spherical liposomes. Multilamellar/multivesicular and unilamellar liposomes are gated in green and magenta, respectively. (F) Image gallery of representative liposomes classified as multilamellar/multivesicular. (G) Image gallery displaying two different phenotypes present in the subpopulation of nonspherical liposomes gated in (B): doublets which correspond to two liposomes attached together (left) and rod-shape liposomes (right). (H) Two-dimensional plot of the *H entropy std* and *H correlation std* features as the outcome of the *feature finder wizard* tool analysis for discriminating between the two subpopulations of nonspherical liposomes shown in (G). (I) Population analysis (*H entropy std* vs *H correlation std*) applied to the nonspherical liposomes. Rod-shaped and doublet liposomes are gated in green and magenta, respectively. (J, K) Graphical representations of the statistics obtained from the morphometric analysis carried out on a set of over 70,000 liposomes from one sample. Nonspherical liposomes that did not belong to the categories “rod shape” or “doublet” were classified as “nonspherical”. For all the images, the white color represents the membrane dye signal. Scale bars are 7 μm.

Quantification of liposome morphological features, such as size, shape, lamellarity, and dispersity, is essential for comparing different methods for liposome production, as well as for understanding the interplay between the inner biochemical processes and membrane mechanics. The histogram of liposome sizes shows a *diameter* of 7.2 ± 1.7 μm (mean and standard deviation), with the majority of the liposomes (91%) having a diameter lower than 10 μm (Figure 3A). The spatial resolution was good enough to clearly distinguish the membrane and subliposomal structures in vesicles bigger than 3 μm in diameter. We then examined the *circularity* feature to quantify any deviation from a spherical shape (Figure 3B,C). The circularity feature, which determines how much the analyzed mask deviates from a circle, is calculated by dividing the average distance between the object’s edge and its center by the variance of such a distance. As a result, the more an object is circular, the smaller the variance and thus the higher the circularity value. We considered liposomes with a circularity higher than 10 as spherical, representing ∼45% of the population of selected liposomes (62,408 events).

To identify multilamellar and multivesicular liposomes within the spherical liposome population we used the IDEAS tool called “*feature finder wizard*”. This tool assists the user at identifying the optimal features that best describe a particular phenotypic trait. To employ the *wizard*, one needs to first select individual event images (at least 25) for each of the phenotypes of interest. From this training data set, the software then suggests the appropriate features that best differentiate the predefined phenotype categories. We manually classified liposomes in two distinct categories, unilamellar and multilamellar, based on visual inspection of some images, and performed the automated analysis. The output graph displayed the *compactness* and *Max Pixel* features (Figure 3D). Compactness measures the degree to which an object is packed together. The higher the value, the more condensed the object. The Max Pixel feature is the largest intensity value obtained from the background subtracted pixels from the input mask. When applying this pair of features to analyze the collection of spherical liposome images, we obtained that ∼58% of the classified vesicles were unilamellar (Figure 3E,J), while the rest exhibited internal membrane structures (Figure 3F). Therefore, from the one million of events collected in total per sample, ∼1.6% (6 × 0.45 × 0.58) corresponds to spherical and unilamellar liposomes, that is 16,000 vesicles. It should be mentioned that, despite the capability of the *wizard* analysis tool to generate two distinct clusters of data points, the method is not perfect and both false positive and false negative events were detected, along with liposomes that were left unclassified. Utilization of the upgraded version of the IDEAS software provided with machine learning algorithms may solve this issue (see Discussion).

We finally sought to classify nonspherical liposomes as rod-shaped or doublets (two liposomes attached together) (Figure 3G), and to quantify their abundance. To distribute liposomes in either of the two categories, we again employed the *feature finder wizard*. The generated data are a scatter plot of the texture features *H-entropy std* and *H-correlation std* (Figure 3H). When applied to the population of nonspherical liposomes, the analysis predicted that ∼15% of liposomes were rod-shaped and ∼69% were doublets (Figure 3I,K).

The results so far demonstrate the power of IFC in acquiring large collections of liposome images and providing statistical population analysis of morphological properties. Next, we applied IFC capabilities for quantitative analysis of synthetic cell modules whose gene-encoded functionalities lead to distinct liposomal phenotypes.

### Lumen Localization Reporter of Transcription–Translation

**Figure 4:**
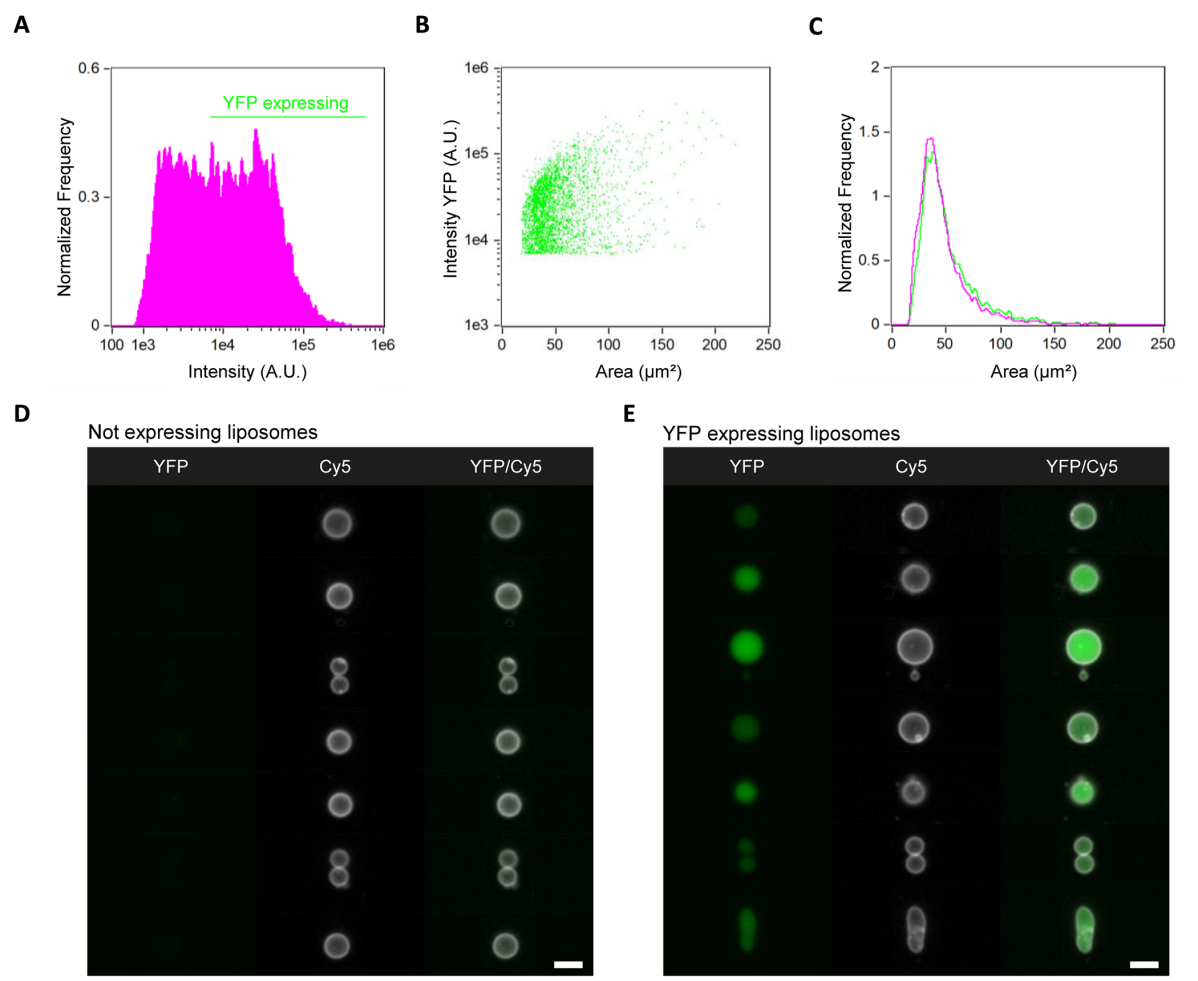
IFC data analysis of the transcription–translation module. (A) Histogram of the internal YFP *intensity* and definition of a threshold to distinguish nonexpressing from gene expressing liposomes. (B) Scatter plot of the liposomes *area* vs the *intensity* of expressed YFP. (C) Comparison of the size distribution between YFP-expressing liposomes (green) and nonexpressing liposomes (magenta). (D, E) Gallery of representative images of nonexpressing liposomes (D) and YFP-expressing liposomes (E). Membrane dye (Cy5) signal is colored in white, YFP is in green. The analysis was performed on a set of over 70,000 liposomes from one sample. Scale bars are 7 μm.

A linear DNA encoding the yellow fluorescent protein (YFP) was expressed in liposomes.^13^ The sample was run into ImageStream and liposomes were identified as described above. To select YFP-expressing liposomes, we generated a fitting mask and plotted the histogram of the *intensity* measured in the 488 nm channel (Figure 4A). After applying an intensity threshold above which liposomes were classified as expressing, we found that ∼60% of liposomes (including nonspherical and multilamellar ones) exhibit YFP signal in their lumen. We estimate the lower limit of detection of freely diffusing YFP in the lumen by IFC to be ∼500 nM for accurate quantitation, similar as we observed with confocal imaging of glass surface-immobilized vesicles.^13^ This corresponds to ∼10,000 molecules in a 4-μm diameter liposome. Intensity values of YFP span across an order of magnitude indicating that transcription–translation efficiency can substantially vary between liposomes. This heterogeneity in gene expression levels holds over the entire range of liposome sizes, with no strong correlation between YFP signal and vesicle size, as shown when plotting the intensity of YFP as a function of the *area* of liposomes (Figure 4B,C). Similar observations have already been reported using confocal fluorescence microscopy.^13^ The main advantages of IFC are that liposomes are imaged in suspension (not in contact to a glass surface), the screening throughput is higher allowing us to perform more accurate statistical population analysis, and that a gallery of individual liposome images is generated in real-time (Figure 4D,E).

### DNA Replication Coupled to Transcription–Translation

We recently designed and implemented in PURE system a self-replicating DNA based on the essential replication proteins of bacteriophage Φ29.^4^ Cell-free expression of the replicator DNA in liposomes yielded exponential amplification of DNA, which led to increased fluorescence of a nucleic acid intercalating dye, as observed by confocal microscopy.^4^ Fluorescence was not always evenly distributed in the vesicle lumen, but localized into bright spots (or replication “blobs”), suggesting aggregation of concentrated DNA in the presence of some PURE compounds (e.g., spermidine, inorganic pyrophosphate, Mg^2+^).^4^ No statistical population analysis was performed in our previous study because of significant background from the acridine orange DNA binding dye and the limited number of imaged liposomes.

**Figure 5:**
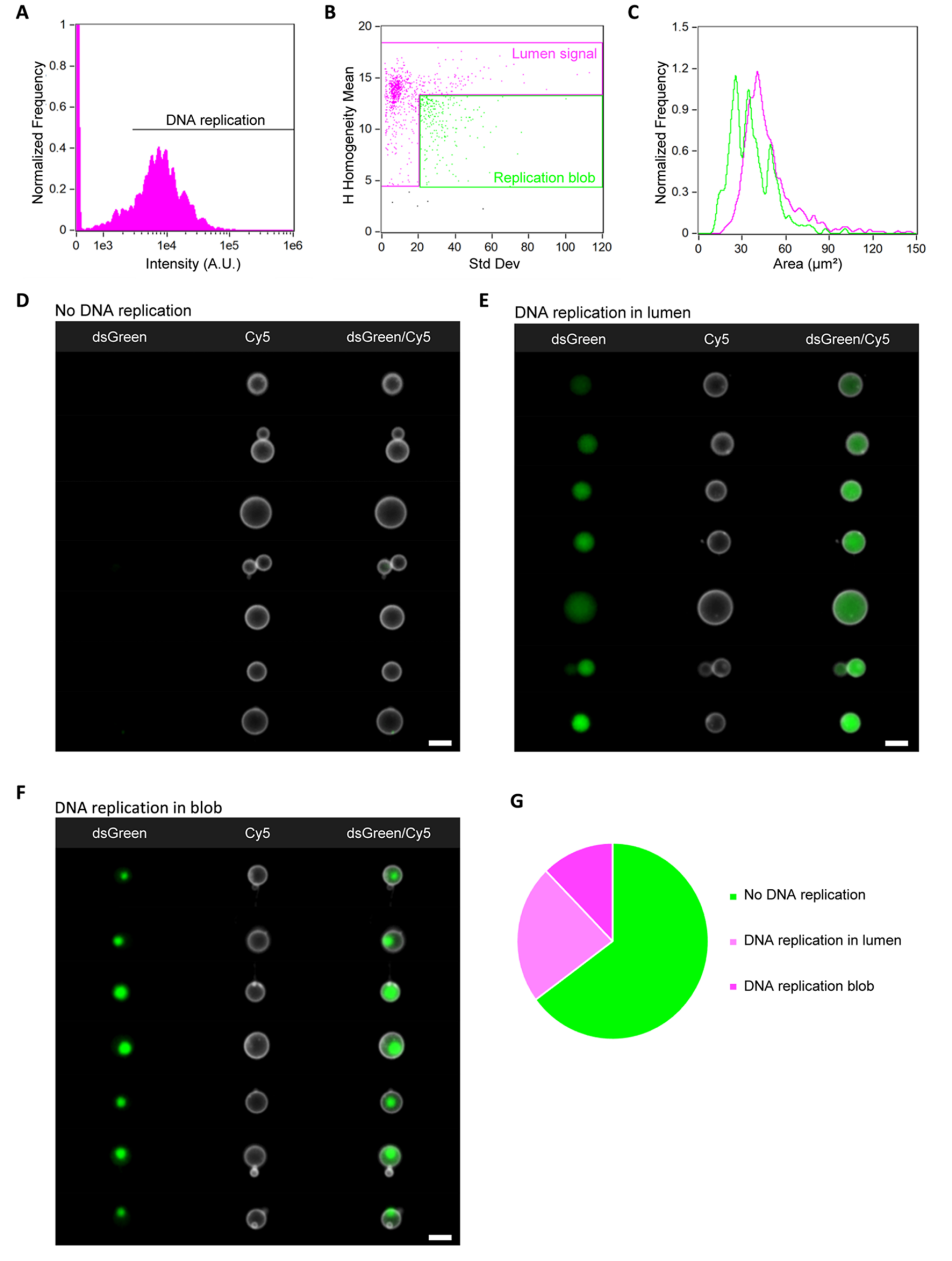
IFC data analysis of the DNA replication module. (A) Histogram of the intensity of the DNA-binding dye dsGreen in liposomes. Successful DNA replication is reflected by high intensity values. (B) Scatter diagram of the *H-homogeneity mean* vs *std dev* features to classify images with respect to the distribution of dsGreen fluorescence in the vesicle lumen. Liposomes with homogeneously distributed dsGreen are gated in magenta, while vesicles exhibiting a “replication blob” are gated in green. (C) Histograms of liposome size (area) for the two subpopulations defined in (B). Color coding is the same as in (B). Liposomes with a replication blob are on average smaller than those with an even intraluminal signal. (D–F) Gallery of representative images of liposomes with no DNA replication (D), increased DNA amount with homogeneous spatial localization (E), and increased DNA amount with formation of condensates (F). (G) Graphical representation of the statistics obtained from the analysis of over 60,000 liposomes from one sample. For all the images, the membrane dye (Cy5) signal is colored in white and DNA-bound dsGreen is in green. Scale bars are 7 μm.

Herein, we exploited the assets of IFC, combined with dsGreen as a lower-background DNA dye, to quantitatively assay in-liposome DNA replication. From the collected single-liposome images, we computed the histogram of dsGreen *intensity* and found that ∼36% of liposomes were active for DNA replication (i.e., dsGreen signal was higher than background-corrected threshold) (Figure 5A). We visually identified three distinct phenotypes based on the intensity and lumen localization of dsGreen fluorescence signal: no or weak dsGreen signal (Figure 5D), homogeneous intensity of dsGreen within the lumen (Figure 5E), and (usually one) replication blob (Figure 5F), the latter two phenotypes corresponding to successful DNA amplification. To distinguish these two visual phenotypes, we screened several IDEAS features and found that *std dev* and *H-homogeneity mean* offered the best combination to discriminate them with high accuracy (Figure 5B). The std dev feature describes the general distribution of pixel intensities by computing the standard deviation in the defined mask. A greater std dev value implies a higher texture. Liposomes with high std dev and low H-homogeneity mean typically display a bright replication blob (Figure 5B), representing ∼34% of the vesicle population (Figure 5G). Moreover, we discovered that the liposomes exhibiting a replication blob had a relatively smaller area (size) compared to those having a homogeneously distributed dsGreen signal (Figure 5C). Further investigations are needed to clarify which factors trigger the formation of DNA condensation. The results demonstrate that IFC is capable to reveal and provide statistical analysis of phenotypic heterogeneity at subliposomal resolution.

### Protein Self-Organization into Bacterial Microtubules

**Figure 6:**
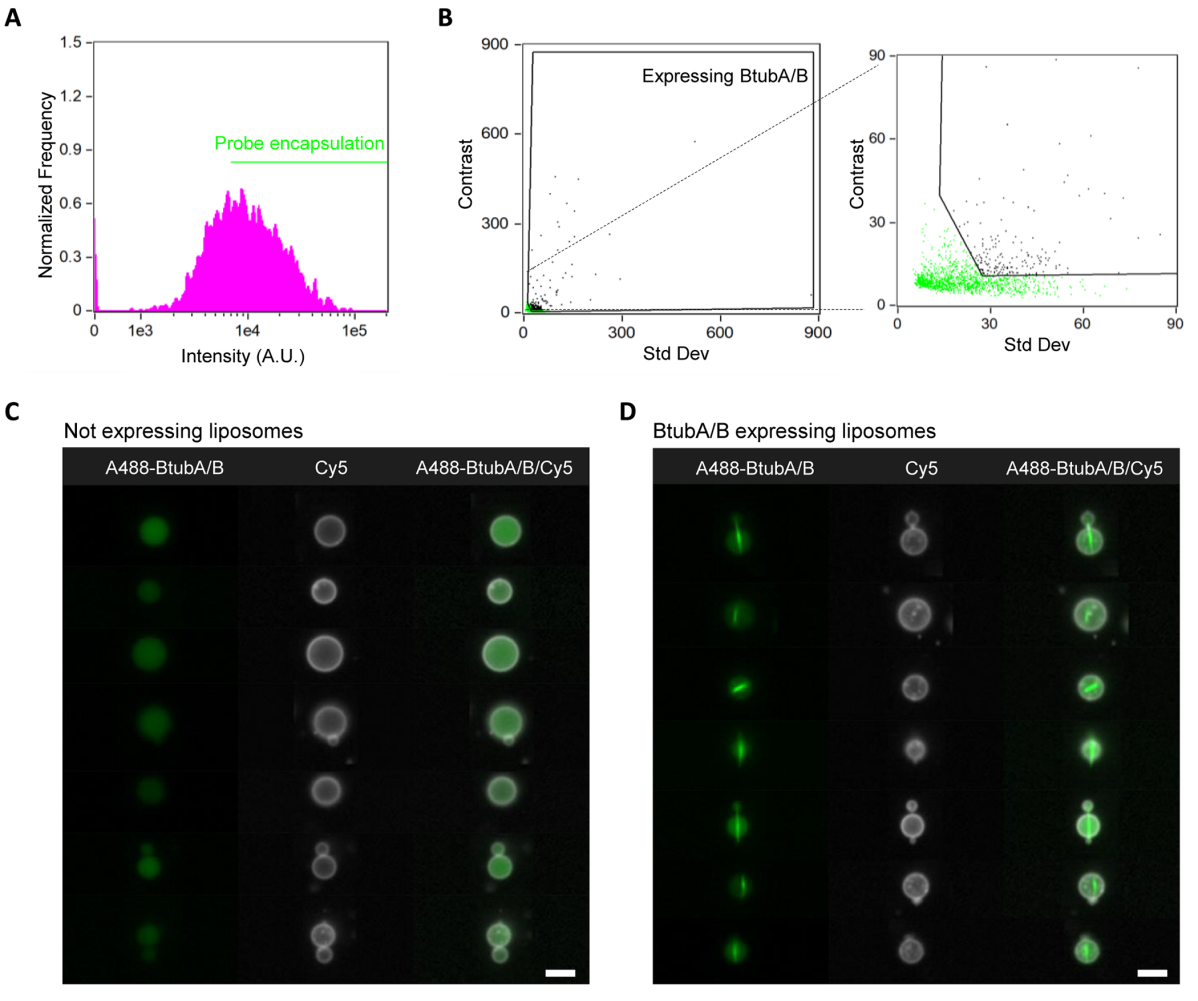
IFC data analysis of the bacterial microtubule module. (A) Histogram of the intensity of encapsulated A488-BtubA/B. Liposomes with an above-threshold intensity value were selected. (B) Liposomes with cytoskeletal structures were identified by plotting the *std dev* vs the *contrast* feature (black gate). A zoom-in image is shown on the side to better visualize the two populations and the applied gating. (C, D) Gallery of representative images of liposomes with encapsulated free tubulin but no filaments (C) or with characteristic microtubules of expressed BtubA/B (D). For all the images, the membrane dye (Cy5) signal is colored in white and A488-BtubA/B is in green. The analysis was performed on a set of over 35,000 liposomes from one sample. Scale bars are 7 μm.

Bacterial microtubules are protein filaments composed of polymerized tubulins BtubA and BtubB from *Prosthecobacter* cells.^24,25^ Expression of the genes *btubA* and *btubB* in PURE system produces BtubA/B microtubules on flat membranes and inside liposomes.^26^ In a synthetic cell, such cytoskeletal structures could play a role in membrane stabilization, polarization or internal trafficking processes. Liposomes with expressed bacterial microtubules were analyzed by IFC. A trace amount of purified BtubA/B labeled with the fluorophore AlexaFluor-488 was coencapsulated for visualization. The membrane signal was employed to select liposomes as described above. We discarded from the analysis all the vesicles that displayed a fluorescence *intensity* value of AlexaFluor-488 lower than a certain threshold (Figure 6A). Then, a wide range of textural features were explored individually or in combination to distinguish the liposomes with protein filaments. The *std dev* function (for detecting inner inhomogeneity) and the *contrast* feature (which assesses an image’s sharpness by identifying big variations in pixel values) proved to be the best combination to detect microtubules, with high std dev and high contrast values (Figure 6B). An image library of the two subpopulations of liposomes with or without self-organized bacterial microtubules is shown in Figure 6C,D. Statistical population analysis revealed that around 4% of liposomes exhibited detectable BtubA/B filaments.

### Relocalization of Min Proteins to the Membrane

The Min system, which comprises the proteins MinC, MinD, and MinE, is primarily responsible for the spatial organization of the division site in *E. coli*.^27^ The Min proteins self-organize at the inner surface of the cytoplasmic membrane and oscillate between the two cell poles in a dynamic manner. MinD and MinE drive the oscillations, while MinC travels on the waves by interacting with MinD.^28^ Reconstitution of Min protein dynamics in liposomes has already been accomplished using PURE system,^7,10^ which may assist binary fission in a prospective synthetic cell.

**Figure 7:**
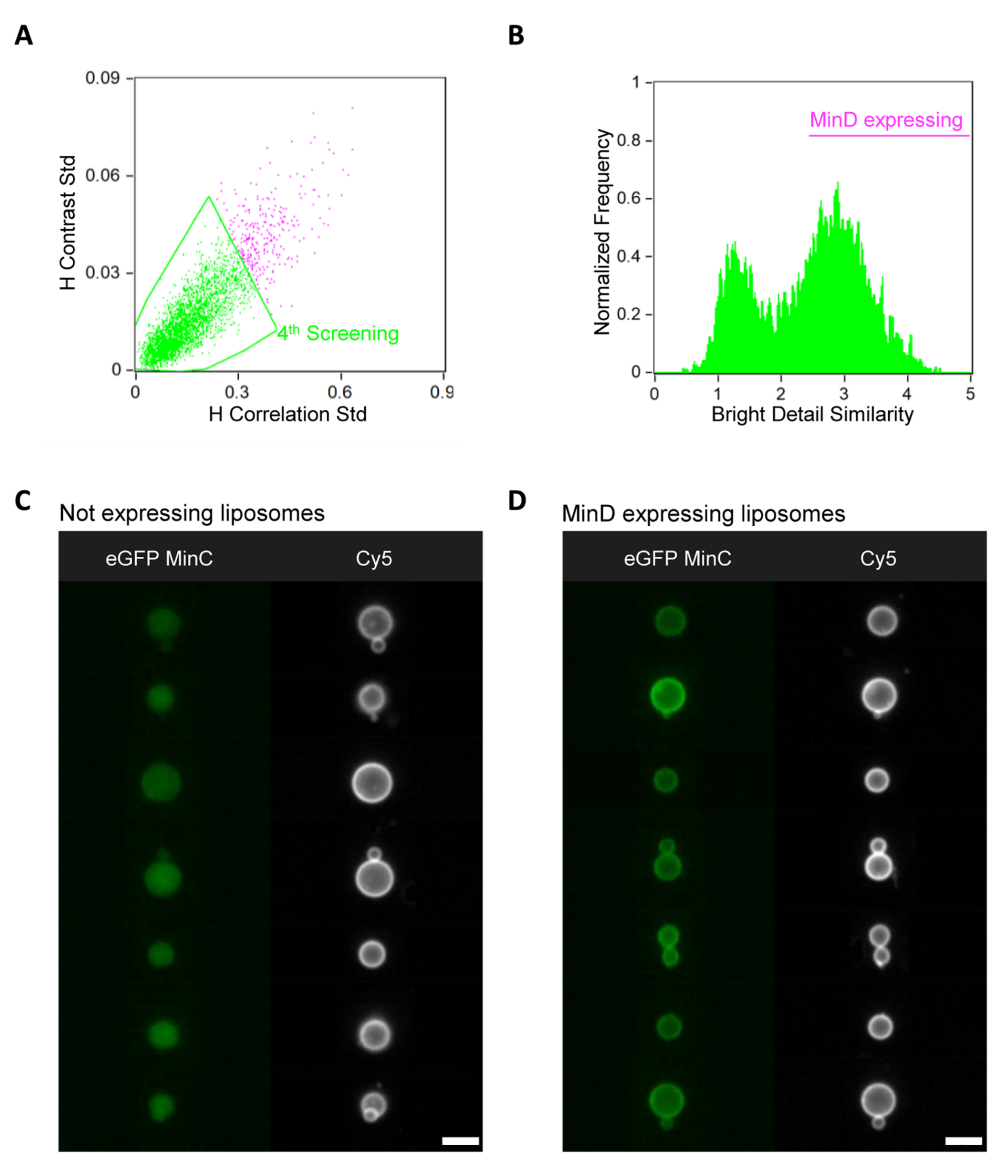
IFC data analysis of the Min self-organization module. (A) Scatter diagram of the parameters *H-correlation std* and *H-contrast std* used as an additional image processing step for the identification of higher quality liposomes. The membrane dye (Cy5) signal was used for the analysis. Gated events considered as good liposomes are colored in green. (B) Histogram of the *bright detail similarity R3* feature (using as inputs the two images in the Cy5 and eGFP-MinC channels) from the liposomes gated in (A). Gated liposomes with active Min proteins exhibit membrane localization of the eGFP-MinC signal upon binding to expressed MinD. (C, D) Gallery of representative images of liposomes with an inactive (lumen localization of eGFP-MinC, panel C) or active (membrane localization of eGFP-MinC, panel D) MinD-MinC system. For all the images, the membrane dye (Cy5) signal is colored in white and eGFP-MinC is in green. The analysis was performed on a set of 40,000 liposomes from one sample. Scale bars are 7 μm.

We challenged IFC to detect the relocalization of eGFP-MinC from the lumen to the membrane, where it is recruited by cell-free expressed MinD. During the liposome identification procedure, we noticed a high percentage of events corresponding to liposomes with inner membrane structures. Thus, we decided to include a more stringent image processing step for accurate selection of unilamellar liposome. A fourth gating using *H-correlation* and *H-contrast standard deviations* in the membrane dye channel was applied to discard events with high local intensity variations (Figure 7A). Next, discrimination of liposomes with eGFP-MinC located exclusively in the lumen or also on the membrane (i.e., expressing MinD), was carried out with the *bright detail similarity R3* feature (Figure 7B). This function compares the bright details of two images and can be used to measure signal colocalization. The feature computes the log-transformed Pearson’s correlation coefficient of small bright regions (3 pixels radius) inside the mask provided for the two input images. Here, the membrane dye and the eGFP-MinC images were chosen as the two inputs. Events with a bright detail similarity greater than 2.5 were considered as correlated (eGFP-MinC also localized at the membrane) and the liposomes were classified as MinD-expressing, which accounted for approximately 54% of the total population. An image gallery of liposomes classified as MinD-expressing or not is displayed in Figure 7C,D.

### PssA-Catalyzed Synthesis of PS Lipid

**Figure 8:**
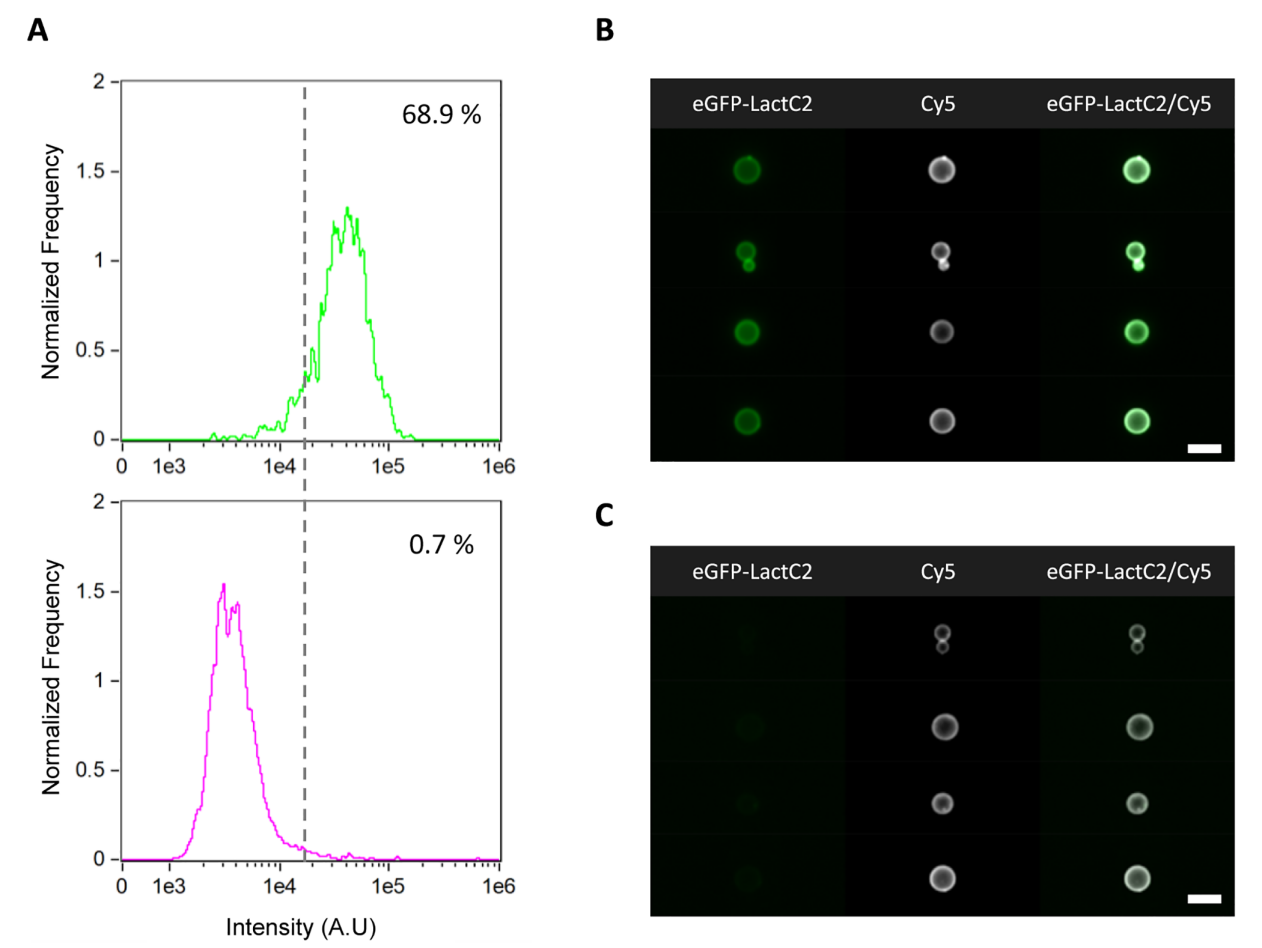
IFC data analysis of the PssA-catalyzed phosphatidylserine biosynthesis module. (A) Histogram of the fluorescence intensity of the liposome membrane-recruited eGFP-LactC2 (PS-specific probe) in a positive sample (+DNA, top) and in a control negative sample (−DNA, bottom). The control sample was run in order to exclude false positive events resulting from mild unspecific binding of the probe. The applied intensity threshold is indicated by the vertical dashed line and the percentage of PS-containing liposomes is appended in each graph. (B, C) Gallery of representative images of PS-producing (B) and nonproducing (C) liposomes. For all the images, the membrane dye (Cy5) signal is colored in white and eGFP-LactC2 signal is in green. The analysis was performed on a set of over 50,000 liposomes from one sample. Scale bars are 7 μm.

The DNA-encoded production and incorporation of membrane constituents is essential for sustainable growth of synthetic cells. Enzymes from the *E. coli* Kennedy pathway for phospholipid biosynthesis were expressed in PURE system and newly synthesized lipids were inserted in the liposome membrane.^29^ One of these enzymes, CDP-diacylglycerol-serine *O*-phosphatidyltransferase (PssA), converts cytidine diphosphate (CDP) lipid headgroup into phosphatidylserine (PS) using l-serine as a cosubstrate. Liposomes containing a small fraction of CDP-DAG lipids in the membrane were formed, the *pssA* gene was expressed with coencapsulated PURE system and l-serine, and PS-containing vesicles were stained using the fluorescent probe eGFP-LactC2 prior analysis with ImageStream. Recruitment of the externally added eGFP-LactC2 to the liposome membrane indicates internal expression of PssA and concomitant production of PS. We assume that flip-flop of PS from the inner to the outer membrane leaflet precedes binding to eGFP-LactC2, a process that may be less energetically unfavorable in PURE-containing liposomes compared to simple buffer conditions. Since we noticed mild unspecific binding of the eGFP-LactC2 probe to the liposome membrane, we decided to also run a negative control sample, where the *pssA* gene was omitted. We plotted the histograms of fluorescence *intensity* of eGFP-LactC2 and defined a threshold value above which liposomes were classified as PS-containing with a minimal number of false positives (Figure 8A). An image gallery of the two subpopulations of liposome images is shown in Figure 8B,C. Under these conditions, about 62% of the liposomes successfully converted CDP-DAG lipids into PS by the internally expressed PssA enzyme.

## Discussion

We introduced IFC for high-throughput imaging of liposomes in laminar flow. The general applicability of IFC in synthetic cell research was demonstrated by assaying diverse populations of liposomes with gene-encoded functional modules. A comprehensive workflow was developed to collect and process up to one million of images of single vesicles, allowing for the evaluation of fluorescence and morphological parameters with statistical analysis of subpopulations, and for sample comparison with no selection bias. The commercial IDEAS software provides over 200 features, covering a wide range of liposome phenotypes that can be quantified. The capability to retrieve images of individual events from plotted data strongly reduces the risks to include false-positive or false-negative events in the analysis.

From the 1 million events detected per sample, about 6% were classified as “good” liposomes (60,000 liposomes on average across all the samples assayed in the study). Besides the stringent gating chosen, it is possible that some liposomes get disrupted during sample filtration before loading to ImageStream or inside the flow device. It is important to note that the samples consisted of a diluted solution of only 1 μL of liposome suspension taken from the 20-μL PURE solution used for lipid film swelling. Scaling up the liposome suspension volume or concentration would straightforwardly increase the number of events, which may be relevant for statistical analysis of very low abundance phenotypes. Data analysis pipelines can easily be saved as templates for the analysis of multiple batches from 1,000 to 100,000 events each. Data processing is then rapid and not excessively computationally demanding, even for complex phenotypes as reported here. To increase the fraction of good liposomes over debris and aggregates, a stricter gating could be performed during image acquisition, for instance by applying the first step (Figure 2B)—or first two steps (Figure 2E)—in our current postacquisition image analysis pipeline. Because the user cannot adjust the channel masks during acquisition, we recommend to perform this step again during offline data analysis anyway.

Further expansion of imaging capabilities with more than two colors (488 and 642 nm laser lines are provided in the basic ImageStream configuration) is also possible with additional wavelengths (405, 561, and 592 nm are also available), which can be useful for multispectral analysis of complex phenotypes. Furthermore, increased analysis power and workflow simplification on the IDEAS software are now possible with a new machine learning module (https://www.luminexcorp.com/imagestreamx-mk-ii/#software).

Many protocols for the formation of giant liposomes have been reported in the literature (we here limit the citations to articles, in which cell-free gene expression was demonstrated),^30−36^ each laboratory often having its preferred method based on available equipment, experimental or biological constraints, inclination to microfluidic approaches or not, etc. It would however be relevant for the synthetic cell community to be offered some guidelines to choose the most appropriate methodology for liposome preparation (including lipid composition and other input parameters) on the basis of objective performance metrics with robust population statistics. In this context, IFC represents a technology of choice for high-throughput screening and quantitative analysis of different liposome samples. The presented workflow provides a generic template for analyzing liposome samples prepared with any methodologies. Other types of synthetic cell chassis, such as peptide vesicles,^37^ polymersomes,^38^ and polymer microcapsules containing a clay-hydrogel,^39^ could also be analyzed with IFC. In that case, users can define other sets of IDEAS features and adjust the gating stringency for some of the steps as this may better fit their purposes.

Leveraging IFC with physical sorting of liposome subpopulations will open the door to directed evolution of synthetic cells.^40^ The recent technological breakthrough in image-activated cell sorting^41^ represents a milestone toward imaging-based selection of liposomes exhibiting desired phenotypic traits, their selection for further analytical investigations, and enrichment of genetic variants conferring a higher degree of aliveness. Finally, as IFC is an on-chip technology, it could be combined with microfluidic production of liposomes, creating a completely automated platform for synthetic cell generation and analysis.

## Methods

### Purified Proteins

eGFP-MinC was purified according to published protocols.^40^ Protein concentration was determined by Bradford assay and by measuring eGFP absorbance. BtubA/B was purified and labeled with AlexaFluor-488 as previously described.^26^ Concentration of purified bacterial tubulin was determined by absorbance measurement at 280 nm (extinction coefficient 103,754.2 M^–1^ cm^–1^). Purified Φ29 DNA-binding proteins were produced as described in refs (41) and (42). Purified eGFP-LactC2 was prepared as described in ref (29).

### DNA Constructs

All DNA templates expressed in PURE system were linear products of polymerase chain reactions (PCR) from a parental plasmid. Constructs containing the *minD*, *btubA*, or *btubB* gene were prepared as previously reported.^10,26^ Forward and reverse primers ChD709 and ChD757, respectively annealing to the T7 promoter and T7 terminator sequences, were used for PCR. The *yfp, pssA*, and *p2-p3* (self-replicating DNA) expressing plasmids were subcloned into ori-containing vectors via Gibson assembly. All the plasmids were cloned into *E. coli* Top10 chemically competent cells. Individual colonies were outgrown in LB/ampicillin (50 μg/mL). Plasmids were extracted using the PURE Yield Plasmid Miniprep kit (Promega) and sent for Sanger sequencing confirmation at Macrogen Europe B.V. Linear fragments were obtained from PCR amplification of the transcription cassette in sequence-verified plasmids using Phusion High-Fidelity DNA Polymerase (NEB) with the forward and reverse primers ChD491 and ChD492, respectively. The amplified PCR fragments were purified using QIAquick PCR purification kit (Qiagen). For the purification of *p2-p3* linear DNA, RNeasy MinElute Cleanup columns (Qiagen) were utilized instead of DNA columns provided with the kit. The general QIAquick manufacturer protocol was modified by having a longer pre-elution buffer drying step (at least 4 min at 10,000*g* with open columns), and a longer column incubation step (at least 5 min) with ultrapure water (20–30 μL of Merck Milli-Q water) prior to the final DNA elution. The purified DNA was quantified by Nanodrop 2000c spectrophotometer (Isogen Life Science) and further analyzed for size and purity with DNA gel electrophoresis. Purified DNA fragments were stored at −20 °C. Sequences of the primers (Table S1) and linear constructs that are original to this study can be found in the Supplementary Methods.

### Lipids

1,2-Dioleoyl-*sn*-glycero-3-phosphocholine (DOPC), 1,2-dioleoyl-*sn*-glycero-3-phosphoethanolamine (DOPE), 1,2-dioleoyl-*sn*-glycero-3phosphoglycerol (DOPG), 1′,3′-bis[1,2-dioleoyl-*sn*-glycero-3-phospho]-glycerol (18:1 cardiolipin), 1,2-distearoyl-*sn*-glycero-3-phosphoethanolamine-*N*-[biotinyl(polyethylene glycol)-2000 (DSPE-PEG-biotin), 1,2-dioleoyl-*sn*-glycero-3-(cytidine diphosphate) (CDP-DAG), and DOPE-Cy5 were from Avanti Polar Lipids.

### Preparation of Lipid-Coated Beads

The glass bead-assisted lipid film swelling method was utilized for liposome production.^34^ Lipid-coated microbeads provide a large lipid film surface area, thus a high yield of liposomes even when starting from microliter swelling solution. Moreover, the method is solvent-free and compatible with a large variety of natural and functionalized lipids. Two different lipid mixtures were prepared. Mixture 1 was used in samples for assaying liposome morphology, YFP, DNA replication, tubulin, and Min protein, and it consisted of DOPC (50 mol %), DOPE (36 mol %), DOPG (12 mol %), 18:1 cardiolipin (2 mol %), DSPE-PEG-biotin (1 mass%), and DOPE-Cy5 (0.5 mass%) for a total mass of 2 mg. Mixture 2 was used in samples for assaying PS biosynthesis and it contained DOPC (47.5 mol %), DOPE (34.2 mol %), DOPG (11.4 mol %), 18:1 cardiolipin (1.9 mol %), 1,2-dioleoyl-*sn*-glycero-3-(cytidine diphosphate) (5 mol %), DSPE-PEG-biotin (1 mass%), and DOPE-Cy5 (0.5 mass%) for a total mass of 2 mg. For both mixtures, lipids dissolved in chloroform were mixed in a round-bottom glass flask. Methanol containing 100 mM rhamnose was added to the lipid solution with a chloroform-to-methanol volume ratio of 2.5:1. Then, 1.5 g of 212–300 μm glass beads (acid washed, Sigma-Aldrich) was poured to the lipid-rhamnose solution, and the organic solvent was removed by rotary evaporation at 200 mbar for 2 h at room temperature, followed by overnight desiccation. Lipid-coated beads were stored under argon at −20 °C until use.

### Production of Gene Expressing Liposomes

Twenty microliters of PURE*frex*2.0 (GeneFrontier, Japan) reaction mixtures were assembled on ice in a 1.5 mL Eppendorf tubes according to the supplier’s recommendations. The exact composition was adjusted to the specific biological module to be reconstituted.

- YFP expression: PURE*frex*2.0 and 4 nM of *yfp* DNA.
- DNA replication: PURE*frex*2.0, 20 mM ammonium sulfate, 300 μM dNTPs, 750 μg mL^–1^ purified P5, 210 μg mL^–1^ purified P6, 1.2 units μL^–1^ of Superase-In RNase inhibitor (Ambion), and 4 nM of *p2-p3* DNA.
- Bacterial tubulin: PURE*frex*2.0, 1 μL DnaK mix (GeneFrontier), 100 nM Atto488-BtubA/B, 3.75 nM of *btubA* and 2.5 nM of *btubB* DNA.
- Min system: PURE*frex*2.0, 1 μL DnaK mix, 2.5 mM ATP, 0.5 μM purified eGFP-MinC, 5 nM of *minD* DNA.
- PS synthesis: PURE*frex*2.0 and 4 nM of *pssA* DNA.

About 10 mg of lipid-coated beads was transferred to the preassembled PURE*frex*2.0 reaction solution and liposomes were formed by natural swelling of the lipid film. The tubes were gently rotated on an automatic tube rotator (VWR) at 4 °C along its axis for 30 min. The samples were then subjected to four freeze–thaw cycles by alternating short incubations in liquid nitrogen and in ice. Using a cut pipet tip, 10 μL of the liposome suspension was harvested by paying attention to not collect glass beads and transferred to a PCR tube, where it was mixed with 1 μL of DNase I (0.07 U μL^–1^) (Thermo Scientific) to prevent gene expression outside liposomes. Samples were incubated in a ThermalCycler (C1000 Touch, Biorad) at 30 °C (for DNA replication) or 37 °C (for all the other conditions) for 2.5–3 h (bacterial tubulin assay), 3 h (Min system assay), or 16 h for the other cellular modules.

### Sample Preparation for IFC Measurements

Liposome solution of 1 μL was diluted in 100 μL of buffer (20 mM HEPES-KOH pH 7.6, 180 mM potassium glutamate, 14 mM magnesium acetate). To remove any remaining glass beads from the liposome suspension, the diluted sample was gently filtered through a cell-strainer cap (35 μm nylon) and collected into 5 mL round-bottom polystyrene test tubes (Falcon). An additional staining step was performed in some samples prior running IFC experiments. To assay DNA replication, dsGreen (Lumiprobe) dye was supplemented at a 1:100,000 dilution factor of the stock concentration. For PS detection, purified eGFP-LactC2 was added to a final concentration of 316 nM. Samples were incubated for 30–60 min at room temperature before loading into ImageStream.

### Acquisition and Analysis of IFC Data

All samples were analyzed with the Amnis ImageStream^X^ Mk II and INSPIRE acquisition software (201.1.0.724) (Luminex Corporation). The following laser power settings were used:

- Morphology analysis, 150 for 488 nm/50 for 642 nm/3 for 785 nm.
- YFP expression, 40 for 488 nm/50 for 642 nm/3 for 785 nm.
- DNA replication, 15 for 488 nm/50 for 642 nm/3 for 785 nm.
- Bacterial tubulin, 120 for 488 nm/40 for 642 nm.
- Min system, 200 for 488 nm/50 for 642 nm/3 for 785 nm.
- PS synthesis, 200 for 488 nm/160 for 642 nm/3 for 785 nm.

The 60× magnification objective was employed, focus was set to automatic mode, and fluidics were set to low speed and high sensitivity. One million events were collected for each sample, which corresponds to an estimated volume of 40 to 50 μL of the diluted liposome suspension. All data were analyzed and displayed with Amnis IDEAS 6.2 analysis software (Luminex). The shown images are representative of the whole sample. Scatterplots are from 100,000 recorded items, as we analyzed 1 million events by opening them in sequential batch mode.

## Supporting Information Available

The sequence of the DNA constructs, the list of primers, and supplementary data about liposome identification using the brightfield signal (PDF)
