# Supplementary material for: Imaging Flow Cytometry for High-Throughput Phenotyping of Synthetic Cells
Source: ACS Synth Biol. 2023 May 8;12(7):2015–28. doi: 10.1021/acssynbio.3c00074 (PMC10367129; doi:10.1021/acssynbio.3c00074)
Supplement: Supplementary file 1 — sb3c00074_si_001.pdf [file sb3c00074_si_001.pdf]

SUPPLEMENTARY INFORMATION

FOR

Imaging flow cytometry for high-throughput phenotyping of synthetic cells

Elisa Godino[1,†], Ana Maria Restrepo Sierra[1,†], Christophe Danelon[1,2,*]

[1]Department of Bionanoscience, Kavli Institute of Nanoscience, Delft University of Technology, 2629HZ Delft, The Netherlands

[2]Toulouse Biotechnology Institute (TBI), Université de Toulouse, CNRS, INRAE, INSA, 31077 Toulouse, France

[†]Equal contribution

*Correspondence

Email: c.j.a.danelon@tudelft.nl or danelon@insa-toulouse.fr

In the sequences below, the following regulatory elements have been highlighted as indicated:

- **T7 promoter**
- T7 terminator
- **vsv terminator**
- phi29 origins of replication

Sequence of *yfp* (5’→3’, complete linear construct)

AAAGTAAGCCCCCACCCTCACATGATACCATTCTCCTAATATCGACATAATCCGTCGATCCTCGGCATACCATGATCAGGGAGGGAAA
CTACTACTTAATATATCAATCTATAGACCTACTAGATAGGTTTGTCAATGAACAACATAAAACGACACAGAATCCCACGTTTTAGCGC
TTCGTCTGTGTCGCATGTGAAAT**TAATACGACTCACTATAGG**GAGACCACAACGGTTTCCCTCTAGAAATAATTTTGTTTAACTTTAA
GAAGGAGATATACATATGCGGGGTTCTCATCATCATCATCATCATGGTATGGCTAGCATGACTGGTGGACAGCAAATGGGTCGGGAT
CTGTACGACGATGACGATAAGGATCCGATGGTTAGCAAAGGCGAAGAACTGTTTACGGGCGTGGTGCCGATTCTGGTGGAACTGGAC
GGCGACGTGAACGGTCACAAATTCAGCGTTTCGGGCGAAGGTGAAGGCGATGCGACCTATGGTAAACTGACGCTGAAATTTATTTGC
ACCACCGGTAAACTGCCGGTGCCGTGGCCGACCCTGGTTACCACGTTTGGTTATGGCCTGCAGTGTTTCGCGCGCTACCCGGATCATA
TGAAACAACACGACTTTTTCAAATCTGCCATGCCGGAAGGTTATGTGCAGGAACGTACGATTTTCTTTAAAGATGACGGCAACTACAA
AACCCGCGCAGAAGTCAAATTTGAAGGTGATACGCTGGTGAACCGTATTGAACTGAAAGGCATCGATTTCAAAGAAGACGGTAATAT
CCTGGGCCATAAACTGGAATACAACTACAACTCCCACAACGTTTACATCATGGCAGATAAACAGAAAAACGGTATCAAAGTCAACTT
CAAAATCCGCCATAACATCGAAGATGGCTCAGTGCAACTGGCTGACCACTACCAGCAAAACACCCCGATCGGTGATGGCCCGGTTCT
GCTGCCGGACAATCATTATCTGAGCTACCAGTCTGCACTGAGTAAAGATCCGAACGAAAAACGTGACCACATGGTCCTGCTGGAATTT
GTGACGGCGGCTGGTATTACGCTGGGCATGGATGAACTGTATAAATGAAAGCTTCCCGGGAAAGTATATATGAGTAAAGATATCGAC
GCAACTGAATGAAATGGTGAAGGACGGGTCCAGGTGTGGCTGCTTCGGCAGTGCAGCTTGTTGAGTAGAGTGTGAGCTCCGTAACTA
GTCGCGTCGATATCCCCGGGCTAGCATAACCCCTTGGGGCCTCTAAACGGGTCTTGAGGGGTTTTTTGCCTCCTATGATTGGTTGTCTT
ATTACCTTACTTCTATTATAGTATAACATGTTAAACGATAGTTTGTCTACCCTTTTCGACAAATTGATGATAATAAATAGTATAGGTAT
ATAGTCGTGATTTAGTTGTTAGATTCTTGTCGAAGATAGTCGGTCAATGGGGAAATGGTGTATGTTGTCGCTGTACCCTACTTT

Sequence of *p2-p3* (5’→3’, complete linear construct)

AAAGTAAGCCCCCACCCTCACATGATACCATTCTCCTAATATCGACATAATCCGTCGATCCTCGGCATACCATGATCAGGGAGGGAAA
CTACTACTTAATATATCAATCTATAGACCTACTAGATAGGTTTGTCAATGAACAACATAAAACGACACAGAATCCCACGTTTTAGCGC
TTCGTCTGTGTCGCATGTGAAAT**TAATACGACTCACTATAGG**GAGACCACAACGGTTTCCCTCTAGAAATAATTTTGTTTAACTTTAA
GAAGGAGATATACATATGCCGCGTAAAATGTACAGCTGCGATTTTGAAACGACGACGAAAGTTGAAGATTGCCGTGTCTGGGCCTAT
GGTTATATGAACATCGAAGACCATTCAGAATATAAAATTGGCAACTCGCTGGATGAATTTATGGCGTGGGTGCTGAAAGTTCAGGCC
GACCTGTACTTCCACAATCTGAAATTTGATGGTGCGTTCATTATCAACTGGCTGGAACGTAATGGCTTTAAATGGAGCGCCGATGGTC
TGCCGAACACCTATAATACGATTATCTCTCGTATGGGCCAATGGTATATGATTGATATCTGCCTGGGCTACAAAGGTAAACGCAAAAT
TCATACCGTGATCTATGACAGCCTGAAAAAACTGCCGTTTCCGGTGAAGAAAATTGCGAAAGATTTCAAACTGACCGTCCTGAAAGG
CGATATTGACTATCACAAAGAACGTCCGGTTGGTTACAAAATCACGCCGGAAGAATATGCGTACATTAAAAACGATATCCAGATTAT
CGCAGAAGCTCTGCTGATTCAGTTTAAACAAGGCCTGGATCGCATGACCGCCGGCAGTGACTCCCTGAAAGGTTTCAAAGATATCATC
ACCACGAAAAAATTTAAGAAAGTGTTCCCGACCCTGAGCCTGGGTCTGGATAAAGAAGTTCGTTATGCATACCGCGGCGGTTTTACGT
GGCTGAACGACCGTTTCAAAGAAAAAGAAATTGGCGAGGGTATGGTCTTTGATGTGAATAGTCTGTATCCGGCTCAGATGTACTCCCG
CCTGCTGCCGTATGGCGAACCGATCGTTTTCGAGGGTAAATATGTCTGGGATGAAGACTACCCGCTGCATATTCAGCACATCCGTTGT
GAATTTGAACTGAAAGAAGGCTATATTCCGACCATTCAAATCAAACGTAGCCGCTTCTATAAGGGTAACGAATACCTGAAAAGCTCT
GGCGGTGAAATCGCAGACCTGTGGCTGAGTAACGTCGATCTGGAACTGATGAAAGAACATTACGATCTGTACAACGTTGAATACATC
TCCGGCCTGAAATTTAAAGCCACCACGGGTCTGTTTAAAGATTTCATTGACAAATGGACCTACATCAAAACCACGTCTGAAGGTGCAA
TCAAACAGCTGGCTAAACTGATGCTGAACAGCCTGTATGGCAAATTTGCATCTAATCCGGATGTTACCGGTAAAGTCCCGTACCTGAA
AGAAAATGGCGCTCTGGGTTTTCGCCTGGGCGAAGAAGAAACCAAAGATCCGGTGTATACGCCGATGGGTGTTTTCATTACCGCGTG
GGCACGTTACACCACCATCACCGCCGCACAAGCGTGCTATGACCGCATTATCTACTGTGATACCGACTCAATTCATCTGACCGGCACG
GAAATCCCGGATGTGATTAAAGATATCGTTGACCCGAAAAAACTGGGTTATTGGGCACACGAATCGACCTTTAAACGTGCTAAATAC
CTGCGCCAGAAAACGTACATCCAAGACATCTACATGAAAGAAGTCGATGGCAAACTGGTGGAAGGTTCACCGGATGACTATACCGAC
ATTAAATTTTCGGTGAAATGCGCCGGCATGACCGATAAAATTAAGAAAGAAGTGACGTTCGAAAATTTCAAAGTGGGTTTCAGTCGC
AAAATGAAACCGAAACCGGTCCAAGTTCCGGGCGGCGTTGTGCTGGTCGATGACACCTTCACGATCAAATAAGAATTGTACTAGAG**T**

ATCTGTTAGTTTTTTTCTACTAGAGTACTAGAG**TATCTGTTAGTTTTTTTC**ATCGGATCCCGGGCCCGTCGACTGC**TAATACGACTC
ACTATAGG**GCCCTCTGGAGACACCAGAGGGTTTACATGTTTATTTGTTTAACTTTAAGAAGGAGATATACTAATGGCACGCAGCCCGC
GCATCCGCATCAAAGATAACGACAAAGCCGAATACGCCCGCCTGGTGAAAAATACGAAAGCTAAAATCGCACGTACCAAGAAAAAA
TATGGCGTGGATCTGACGGCTGAAATTGACATCCCGGATCTGGACTCATTTGAAACCCGCGCGCAGTTCAATAAATGGAAAGAACAA
GCTAGCTCTTTTACGAACCGTGCGAATATGCGCTATCAGTTCGAGAAAAACGCCTACGGTGTGGTTGCATCGAAAGCTAAAATTGCGG
AAATCGAACGTAACACCAAAGAAGTTCAACGCCTGGTCGATGAAAAAATCAAAGCCATGAAAGACAAAGAATATTACGCAGGCGGT
AAACCGCAGGGCACGATTGAACAACGTATCGCCATGACCTCACCGGCACATGTGACGGGTATCAACCGTCCGCACGATTTTGACTTCA
GTAAAGTTCGCAGTTACTCCCGTCTGCGCACCCTGGAAGAATCCATGGAAATGCGCACGGATCCGCAGTATTACGAAAAGAAAATGA
TTCAGCTGCAACTGAATTTTATCAAAAGCGTCGAAGGCTCATTTAACTCGTTCGATGCGGCCGACGAACTGATTGAAGAACTGAAGAA
AATTCCGCCGGATGACTTTTATGAACTGTTCCTGCGTATTAGCGAAATCTCTTTTGAAGAATTCGATTCTGAAGGCAACACCGTCGAA
AATGTGGAGGGTAACGTTTACAAAATTCTGTCGTATCTGGAACAATATCGTCGTGGTGATTTTGATCTGTCGCTGAAAGGCTTCTAAT
AGCATAACCCCTTGGGGCCTCTAAACGGGTCTTGAGGGGTTTTTTGCCTCCTATGATTGGTTGTCTTATTACCTTACTTCTATTATAGTA
TAACATGTTAAACGATAGTTTGTCTACCCTTTTCGACAAATTGATGATAATAAATAGTATAGGTATATAGTCGTGATTTAGTTGTTAGA
TTCTTGTCGAAGATAGTCGGTCAATGGGGAAATGGTGTATGTTGTCGCTGTACCCTACTTT

Sequence of *btubA* (5’→3’, complete linear construct)

CAGTCACGACGTTGTAAAACGACGGCCAGTCGCGAAAT**TAATACGACTCACTATAGG**GGAATTGTGAGCGGATAACAATTCCCCTC
TAGAAATAATTTTGTTTAACTTTAAGAAGGAGATATACATATGAAAGTTAATAATACAATTGTAGTTAGTATTGGTCAGGCGGGCAAC
CAAATCGCGGCGAGCTTCTGGAAAACCGTGTGCCTGGAGCACGGTATTGACCCGCTGACCGGTCAGACCGCGCCGGGCGTTGCGCCG
CGTGGTAACTGGAGCAGCTTCTTTAGCAAGCTGGGCGAGAGCAGCAGCGGTAGCTACGTGCCGCGTGCGATCATGGTTGATCTGGAA
CCGAGCGTGATTGACAACGTTAAAGCGACCAGCGGCAGCCTGTTCAACCCGGCGAACCTGATTAGCCGTACCGAGGGCGCGGGTGGC
AACTTTGCGGTTGGTTACCTGGGTGCGGGTCGTGAGGTGCTGCCGGAAGTTATGAGCCGTCTGGATTATGAAATCGACAAGTGCGATA
ACGTGGGTGGCATCATTGTTCTGCATGCGATCGGTGGTGGCACCGGCAGCGGTTTTGGCGCGCTGCTGATCGAGAGCCTGAAGGAAA
AATACGGCGAGATTCCGGTGCTGAGCTGCGCGGTTCTGCCGAGCCCGCAGGTGAGCAGCGTGGTTACCGAGCCGTATAACACCGTTTT
TGCGCTGAACACCCTGCGTCGTAGCGCGGATGCGTGCCTGATCTTCGATAACGAAGCGCTGTTTGACCTGGCGCACCGTAAATGGAAC
ATTGAGAGCCCGACCGTGGACGATCTGAACCTGCTGATCACCGAAGCGCTGGCGGGCATTACCGCGAGCATGCGTTTCAGCGGTTTTC
TGACCGTGGAAATCACCCTGCGTGAGCTGCTGACCAACCTGGTTCCGCAACCGAGCCTGCACTTCCTGATGTGCGCGTTTGCGCCGCT
GACCCCGCCGGATCGTAGCAAGTTCGAGGAACTGGGTATCGAGGAAATGATTAAAAGCCTGTTCGACAACGGCAGCGTGTTTGCGGC
GTGCAGCCCGATGGAAGGTCGTTTTCTGAGCACCGCGGTTCTGTATCGTGGCATCATGGAGGATAAACCGCTGGCGGATGCGGCGCTG
GCGGCGATGCGTGAAAAGCTGCCGCTGACCTACTGGATTCCGACCGCGTTCAAAATTGGCTATGTTGAGCAGCCGGGTATTAGCCACC
GTAAAAGCATGGTGCTGCTGGCGAACAACACCGAAATCGCGCGTGTTCTGGATCGTATTTGCCACAACTTCGACAAGCTGTGGCAAC
GTAAAGCGTTTGCGAACTGGTATCTGAACGAGGGTATGAGCGAGGAACAGATCAACGTGCTGCGTGCGAGCGCGCAAGAACTGGTGC
AGAGCTATCAAGTTGCGGAGGAAAGCGGCGCGAAGGCGAAAGTTCAAGACAGCGCGGGTGATACCGGTATGCGTGCGGCGGCGGCG
GGTGTGAGCGACGATGCGCGTGGTAGCATGAGCCTGCGTGACCTGGTTGATCGTCGTCGTTAAGCGATCACTAGCATAACCCCTTGGG
GCCTCTAAACGGGTCTTGAGGGGTTTTTTGGGCGTAATCATGGTCATAGCTGTTTCCTGTGTG

Sequence of *btubB* (5’→3’, complete linear construct)

CAGTCACGACGTTGTAAAACGACGGCCAGTCGCGAAAT**TAATACGACTCACTATAGG**GGAATTGTGAGCGGATAACAATTCCCCTC
TAGAAATAATTTTGTTTAACTTTAAGAAGGAGATTTGAAAATGAGAGAAATATTAAGTATACATGTAGGTCAATGCGGCAACCAGAT
CGCGGATAGCTTTTGGCGTCTGGCGCTGCGTGAACACGGCCTGACCGAGGCGGGCACCCTGAAGGAAGGTAGCAACGCGGCGGCGAA
CAGCAACATGGAAGTGTTCTTCCACAAGGTTCGTGACGGTAAATACGTGCCGCGTGCGGTGCTGGTTGATCTGGAGCCGGGCGTTATC
GCGCGTATTGAAGGTGGCGACATGAGCCAGCTGTTTGATGAAAGCAGCATCGTGCGTAAAATCCCGGGTGCGGCGAACAACTGGGCG
CGTGGTTATAACGTGGAGGGCGAAAAAGTTATCGACCAGATTATGAACGTGATCGATAGCGCGGTTGAGAAGACCAAAGGTCTGCAA
GGCTTCCTGATGACCCATAGCATCGGTGGCGGTAGCGGCAGCGGTCTGGGCAGCCTGATTCTGGAACGTCTGCGTCAGGCGTACCCGA
AGAAACGTATCTTCACCTTTAGCGTGGTTCCGAGCCCGCTGATTAGCGACAGCGCGGTGGAGCCGTATAACGCGATCCTGACCCTGCA
ACGTATTCTGGACAACGCGGATGGTGCGGTTCTGCTGGACAACGAAGCGCTGTTCCGTATCGCGAAGGCGAAACTGAACCGTAGCCC
GAACTACATGGATCTGAACAACATCATTGCGCTGATTGTGAGCAGCGTTACCGCGAGCCTGCGTTTTCCGGGCAAGCTGAACACCGAT
CTGAGCGAGTTCGTGACCAACCTGGTTCCGTTCCCGGGCAACCACTTTCTGACCGCGAGCTTCGCGCCGATGCGTGGTGCGGGTCAGG
AAGGTCAAGTGCGTACCAACTTTCCGGACCTGGCGCGTGAAACCTTTGCGCAGGACAACTTCACCGCGGCGATCGATTGGCAGCAAG

GTGTTTATCTGGCGGCGAGCGCGCTGTTCCGTGGCGATGTGAAGGCGAAAGACGTTGATGAAAACATGGCGACCATTCGTAAGAGCC
TGAACTACGCGAGCTATATGCCGGCGAGCGGCGGTCTGAAACTGGGCTATGCGGAAACCGCGCCGGAAGGTTTTGCGAGCAGCGGCC
TGGCGCTGGTGAACCACACCGGTATCGCGGCGGTTTTCGAGCGTCTGATCGCGCAATTCGACATTATGTTTGATAACCACGCGTACAC
CCACTGGTATGAAAACGCGGGTGTTAGCCGTGACATGATGGCGAAAGCGCGTAACCAGATTGCGACCCTGGCGCAGAGCTATCGTGA
TGCGAGCTAAGCAATAACTAGCATAACCCCTTGGGGCCTCTAAACGGGTCTTGAGGGGTTTTTTGGGCGTAATCATGGTCATAGCTGT
TTCCTGTGTG

Sequence of *minD* (5'→3', complete linear construct)

TAATACGACTCACTATAGGGGAATTGTGAGCGGATAACAATTCCCCTCTAGAAATAATTTTGTTTAACTTTAAGAAGGAGATATACA
TATGGCACGCATTATTGTTGTTACTTCGGGCAAAGGGGGTGTTGGTAAGACAACCTCCAGCGCGGCCATCGCCACTGGTTTGGCCCAG
AAGGGAAAGAAAACTGTCGTGATAGATTTTGATATCGGCCTGCGTAATCTCGACCTGATTATGGGTTGTGAACGCCGGGTCGTTTACG
ATTTCGTCAACGTCATTCAGGGCGATGCAACGCTAAATCAGGCGTTAATTAAAGATAAGCGTACTGAAAATCTCTATATTCTGCCGGC
ATCGCAAACACGCGATAAAGATGCCCTCACCCGTGAAGGGGTCGCCAAAGTTCTTGATGATCTGAAAGCGATGGATTTTGAATTTATC
GTTTGTGACTCCCCGGCAGGGATTGAAACGGTGCGTTAATGGCACTCTATTTTGCAGACGAAGCCATTATTACCACCAACCCGGAAGT
CTCCTCAGTACGCGACTCTGACCGTATTTTAGGCATTCTGGCGTCGAAATCACGCCGCGCAGAAAATGGCGAAGAGCCTATTAAAGAG
CACCTGCTGTTAACGCGCTATAACCCAGGCCGCGTAAGCAGAGGTGACATGCTGAGCATGGAAGATGTGCTGGAGATCCTGCGCATC
AAACTCGTCGGCGTGATCCCAGAGGATCAATCAGTATTGCGCGCCTCTAACCAGGGTGAACCGGTCATTCTCGACATTAACGCCGATG
CGGGTAAAGCCTACGCAGATACCGTAGAACGTCTGTTGGGAGAAGAACGTCCTTTCCGCTTCATTGAAGAAGAGAAGAAAGGCTTCC
TCAAACGCTTGTTCGGAGGATAAGGATCCGGCTGCTAACAAAGCCCGAAAGGAAGCTGAGTTGGCTGCTGCCACCGCTGAGCAATAA
CTAGCATAACCCCTTGGGGCCTCTAAACGGGTCTTGAGGGGTTTTTTG

Sequence of *pssA* (5'→3', complete linear construct)

AAAGTAAGCCCCCACCCTCACATGATACCATTCTCCTAATATCGACATAATCCGTCGATCCTCGGCATACCATGATCAGGGAGGGAAA
CTACTACTTAATATATCAATCTATAGACCTACTAGATAGGTTTGTCAATGAACAACATAAAACGACACAGAATCCCACGTTTTAGCGC
TTCGTCTGTGTCGCATGTGAAAT**TAATACGACTCACTATAGG**GGAATTGTGAGCGGATAACAATTCCCCTCTAGAAATAATTTTGTTT
AACTTTAAGAAGGAGATATACATATGGCTAGCATGACTGGTGGACAGCAAATGGGTCGCGGATCCGGCTGCATGTTGTCAAAATTTA
AGCGTAATAAACATCAACAACACCTTGCCCAACTACCCAAGATTTCTCAATCAGTTGATGATGTCGATTTCTTTTACGCTCCCGCCGAC
TTCCGGGAGACGCTGCTGGAAAAAATAGCCAGCGCGAAGCAGCGCATTTGCATTGTCGCCCTGTATCTCGAACAGGATGACGGTGGC
AAAGGCATTCTGAACGCGTTGTATGAGGCTAAAAGGCAGCGTCCGGAACTGGATGTGCGGGTGCTGGTCGACTGGCATCGTGCACAA
CGTGGACGCATTGGCGCTGCGGCATCTAACACTAACGCTGACTGGTACTGCCGCATGGCGCAGGAAAATCCGGGCGTAGATGTTCCG
GTTTATGGCGTTCCAATCAATACTCGTGAAGCCCTTGGTGTTCTGCACTTTAAAGGCTTTATCATCGACGATAGCGTACTTTATAGCGG
TGCCAGCCTGAACGATGTTTACCTGCATCAGCACGATAAATATCGCTACGACCGTTATCATCTGATCCGTAACCGTAAGATGTCAGAC
ATTATGTTTGAATGGGTTACACAGAATATTATGAATGGCCGCGGCGTTAATCGTCTGGATGATGTTAATCGGCCAAAAAGCCCGGAAA
TCAAGAACGATATTCGTCTGTTCCGCCAGGAGCTGCGTGATGCCGCTTATCATTTCCAGGGCGATGCCGACAACGATCAGCTTTCTGT
AACGCCGCTAGTGGGGCTGGGGAAATCGAGTCTGTTGAACAAGACCATTTTCCATCTTATGCCTTGTGCCGAGCAGAAACTAACCATC
TGTACGCCATACTTCAACCTGCCAGCAATCCTTGTGCGCAATATTATCCAGTTGCTGCGCGAAGGGAAAAAGGTCGAAATTATTGTTG
GTGATAAAACCGCGAATGACTTCTACATTCCGGAAGATGAACCTTTCAAGATAATTGGCGCATTGCCTTATCTCTATGAGATCAATCT
GCGTCGTTTCCTGAGCCGTTTGCAGTATTACGTCAATACTGACCAGCTAGTGGTTCGGTTATGGAAAGATGACGACAACACCTATCAC
CTGAAAGGGATGTGGGTTGATGATAAGTGGATGTTGATCACCGGTAATAACCTGAACCCGCGCGCCTGGCGTCTGGATCTGGAAAAC
GCCATTTTGATCCACGATCCGCAACTTGAGCTGGCGCCACAGCGAGAGAAAGAACTGGAGCTGATCCGCGAGCATACCACCATCGTT
AAGCACTATCGCGATCTGCAAAGTATTGCCGATTATCCGGTGAAGGTTCGTAAACTCATCCGCCGTTTGCGCCGTATCCGCATCGACC
GATTAATTAGCCGCATCCTGTAACAAAGCCCGAAAGGAAGCTGAGTTGGCTGCTGCCACCGCTGAGCAATAATAGCATAACCCCTTG
GGGCCTCTAAACGGGTCTTGAGGGGTTTTTTGCCTCCTATGATTGGTTGTCTTATTACCTTACTTCTATTATAGTATAACATGTTAAACG
ATAGTTTGTCTACCCTTTTCGACAAATTGATGATAATAAATAGTATAGGTATATAGTCGTGATTTAGTTGTTAGATTCTTGTCGAAGAT
AGTCGGTCAATGGGGAAATGGTGTATGTTGTCGCTGTACCCTACTTT

Table S1: List of primers used in this study

Name	Sequence (5'→ 3')
ChD491	AAAGTAAGCCCCCACCCTCACATG
ChD492	AAAGTAGGGTACAGCGACAACATACAC
ChD709	CAAAAAAACCCCTCAAGACCCGTTTAGAGG
ChD757	TAATACGACTCACTATAGGG

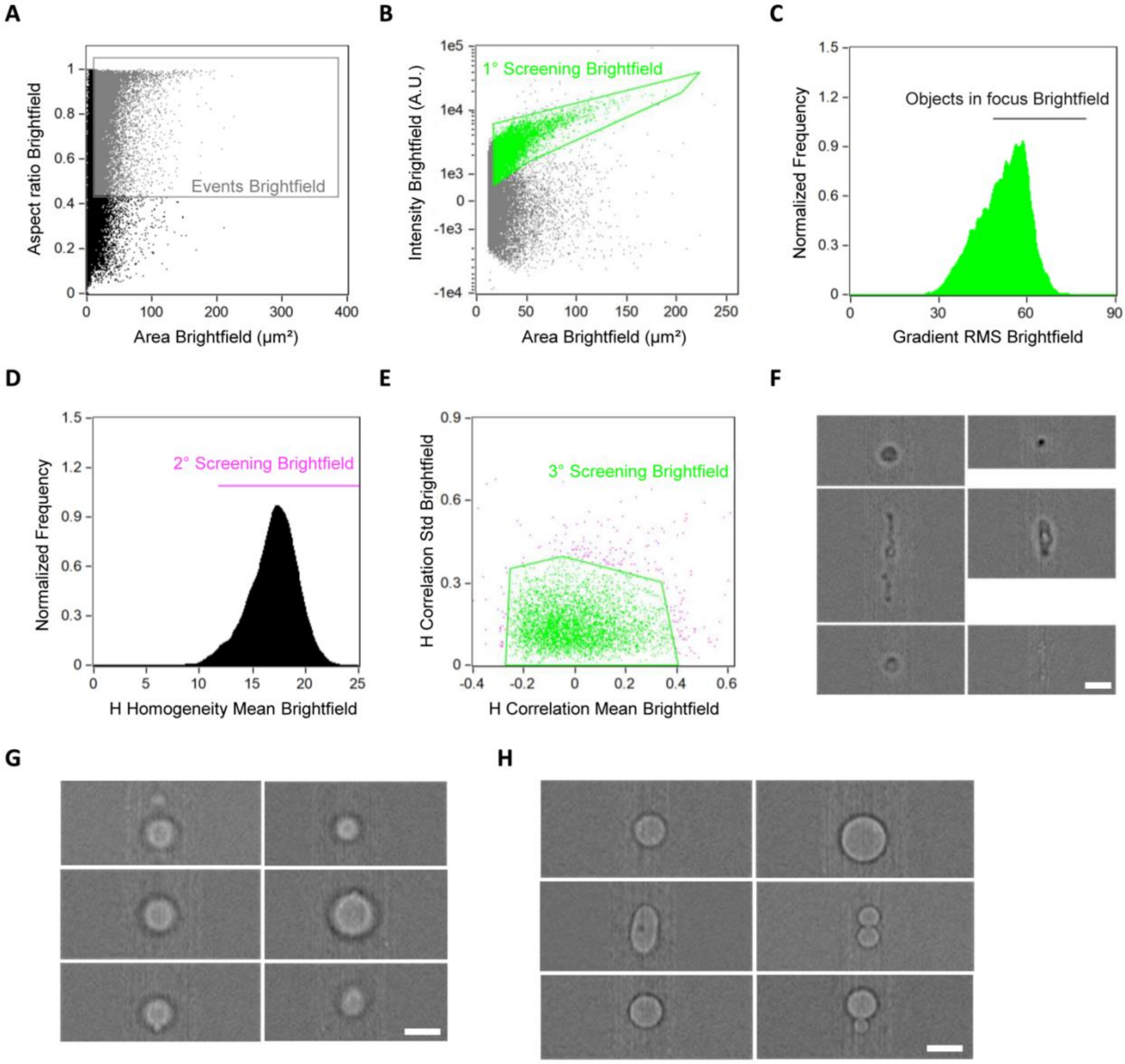

Figure S1: Liposome identification using the brightfield channel. The brightfield channel was used to identify liposome that did not have a membrane signal. The pipeline is similar to the one reported in Figure 2. A mask fitting the area to be used during the analysis in the brightfield channel was created. Identifying liposomes via the brightfield channel was more challenging than using a membrane dye signal. Structural characterization was less precise and stricter cut-off values were needed for the selection of actual liposomes. This stringency can, however, discard some "good" liposomes. We always recommend visual inspection of all gated populations for validation. **A)** Scatter diagram of the *area* of the mask and *aspect ratio* features. Objects having a surface area > 30 μm^2 and an aspect ratio > 0.4 were gated as relevant events (grey gate). **B)** Scatter plot of the *intensity* of the brightfield signal and the *area* of the mask (green gate). **C)** In-focus events were selected based on the *gradient RMS* feature. The events having a gradient RMS > 45 were selected for further analysis (black gate). **D)** Selection of liposomes with high *H-homogeneity* values (magenta gate). **E)** Scatter diagram of the *H-Correlation mean* and *standard deviation*. The selected objects (green gate) correspond to the final liposome population, which is cleared from undesired events. **F)** Images of debris, aggregates with different sizes and shapes that were eliminated. **G)** Images of out-of-focus liposomes that were excluded from the analysis. **H)** Gallery of liposome images that passed the gating steps. Analysis in the brightfield channel was performed on a small set of 8,000 liposomes from one sample. Scale bars are 7 µm.
